# A systematic review of cardiac *in-silico* clinical trials

**DOI:** 10.1088/2516-1091/acdc71

**Published:** 2023-06-22

**Authors:** Cristobal Rodero, Tiffany M G Baptiste, Rosie K Barrows, Hamed Keramati, Charles P Sillett, Marina Strocchi, Pablo Lamata, Steven A Niederer

**Affiliations:** 1 Cardiac Electro-Mechanics Research Group (CEMRG), National Heart and Lung Institute, Imperial College London, London, United Kingdom; 2 Cardiac Electro-Mechanics Research Group (CEMRG), Department of Biomedical Engineering and Imaging Sciences, King’s College London, London, United Kingdom; 3 Cardiac Modelling and Imaging Biomarkers (CMIB), Department of Biomedical Engineering and Imaging Sciences Department, King’s College London, London, United Kingdom; 4 Turing Research and Innovation Cluster in Digital Twins (TRIC: DT), The Alan Turing Institute, London, United Kingdom

**Keywords:** computational cardiology, computational trial, virtual clinical trials, heart simulation, digital twin

## Abstract

Computational models of the heart are now being used to assess the effectiveness and feasibility of interventions through *in-silico* clinical trials (ISCTs). As the adoption and acceptance of ISCTs increases, best practices for reporting the methodology and analysing the results will emerge. Focusing in the area of cardiology, we aim to evaluate the types of ISCTs, their analysis methods and their reporting standards. To this end, we conducted a systematic review of cardiac ISCTs over the period of 1 January 2012–1 January 2022, following the preferred reporting items for systematic reviews and meta-analysis (PRISMA). We considered cardiac ISCTs of human patient cohorts, and excluded studies of single individuals and those in which models were used to guide a procedure without comparing against a control group. We identified 36 publications that described cardiac ISCTs, with most of the studies coming from the US and the UK. In $75\%$ of the studies, a validation step was performed, although the specific type of validation varied between the studies. ANSYS FLUENT was the most commonly used software in $19\%$ of ISCTs. The specific software used was not reported in $14\%$ of the studies. Unlike clinical trials, we found a lack of consistent reporting of patient demographics, with $28\%$ of the studies not reporting them. Uncertainty quantification was limited, with sensitivity analysis performed in only $19\%$ of the studies. In $97\%$ of the ISCTs, no link was provided to provide easy access to the data or models used in the study. There was no consistent naming of study types with a wide range of studies that could potentially be considered ISCTs. There is a clear need for community agreement on minimal reporting standards on patient demographics, accepted standards for ISCT cohort quality control, uncertainty quantification, and increased model and data sharing.

## 1. Introduction

According to the National Institute of Health, clinical trials are research studies in which one or more human subjects are prospectively assigned to one or more interventions (which may include placebo or other control) to evaluate the effects of those interventions on health-related biomedical or behavioural outcomes^5^ 5
https://grants.nih.gov/policy/clinical-trials/definition.htm.. This definition can be extended to *in-silico* clinical trials (ISCTs), also known as computational or virtual clinical trials [38] as follows. We define an ISCT as a research study that uses computer models of cells, tissues, organs, or systems of human subjects, assigned to one or more interventions (which may include some form of control group) to evaluate the effects of those interventions on health-related biomedical or behavioural outcomes.

Areas within biomedical engineering outside of cardiovascular sciences have been doing ISCTs over the last few years. Recently, ISCTs have been performed to assist the development of a COVID-19 vaccine using agent-based models [50]. Other examples of ISCTs include testing the safety and effectiveness of artificial pancreas [57] or the repurposing of antipsychotic drugs to treat Alzheimer’s disease [23]. Not only exemplary cases, but some authors such as [21] designed a platform to perform ISCTs, aiming for an improvement in the translational aspect of simulations and to bridge the gap between simulations and clinical validation through randomised clinical trials. All these examples showcase how ISCTs have an important role to play in the acceleration of medical therapy/device development and regulation.

In cardiovascular sciences, this methodology could be thought of as a natural extension of the concept of a ‘digital twin’. The digital twin in cardiology is a concept that has spread in recent years in the engineering and clinical communities [8]. However, in the case of ISCTs, although we can find early results, their prevalence, scale, methods, and limitations are not summarised in a single place.

The main purpose of this systematic review of ISCTs in cardiology (referred to as ISCTs throughout the rest of the text to improve readability) is three-fold; first, we explore the types of ISCTs performed; second, we evaluate the analysis methods; and third, we examine the reporting standards. In section 2, we explain how the search, selection, and screening processes were performed; section 3 provides a summary of the selected articles; section 4 discusses the questions raised in section 3, and the main takeaways are summarised in section 5.

## 2. Methods

### 2.1. Study design

We performed a systematic scoping review following the PRISMA 2020 guideline [37]. In this type of review, rather than looking for evidence to answer a specific question, we looked for research done around a specific topic, in this case ISCT in cardiology. The time frame was established to cover any article published in a peer-reviewed journal from 1 January 2012 to 1 January 2022.

### 2.2. Database search strategy

The articles reviewed were identified by a comprehensive electronic search from the Scopus and Pubmed databases. Essentially, we searched for publications that link computational research, cardiac sciences, and trials. Keyword search was performed in titles, abstracts, or keywords. Details on the specific keywords can be found in the supplementary material.

### 2.3. Abstract screening

The results of the literature search were included and analysed using the open access CADIMA software [20]. At the abstract level, a text was discarded if the answer was ‘no’ to any of the following questions:

Q1 Do the authors run cardiovascular simulations on human data?
Q2 If the answer to Q1 is yes, are they modelling a clinical intervention, product, or measurement?

In the case where there was not enough information on the title and abstract only to answer Q1 or Q2, the answer was marked as ‘unsure’ and that text was moved to full text screening.

To ensure that all review authors had the same understanding of the inclusion and exclusion criteria, we performed a consistency check on the abstract and full text levels. In case of disagreement, the criteria were reformulated to improve understanding. We only considered original research articles published in peer-reviewed journals. All review papers, letters, editorials, media articles, conference abstracts, and articles published in a non-peer-reviewed journals and online repositories (such as arXiV) were excluded.

### 2.4. Full-text screening

After completion of the title and abstract screening, the accepted articles moved to the full-text screening level. At this level, a text was discarded if the answer was ‘no’ to any of the following questions:

Q1 Do the authors run cardiovascular simulations using human clinical data?
Q2 Do the authors model a clinical intervention? These interventions may be medical products, such as drugs or devices; or procedures.
Q3 Is there any kind of mechanistic modelling?
Q4 Is it from a peer-reviewed journal?
Q5 Is it from before 2022?
Q6 Do the authors compare the outcome of the intervention with and without the intervention, or with a standard intervention? These interventions may be medical products, such as drugs or devices; or procedures. The comparison might be done in the same population or in different cohorts.

Q1 was modified from the abstract level Q1 to avoid cases where estimations not based on patients were used, rather than patient-specific data. Q5 was added as a quality check to avoid papers published on 2022 where the metadata did not specify the day and month of publication. A consistency check was also performed for the full text level, and the questions were clarified for the cases where there was a disagreement.

A paper was also excluded if the full text was not available, if it was duplicated, if no primary data were presented, or if it was not assessable. A paper was considered not assessable if it was written in a language that was not readable by any of the reviewers. The languages readable by the authors were English, Spanish, Persian, and Italian.

Once the articles were screened, five types of questions were answered about each article for a total of 16 questions: 2 questions about the authors, 4 about the population, 4 about the resources used, 3 about the model(s) used, and 3 about the study in general.

The CADIMA pipeline is openly available at www.cadima.info/ under the same title of this article. The scripts to reproduce the figures of this review can be found at github^6^ 6
https://github.com/CEMRG-publications/Rodero_2023_ProgrBiomedEng.. Details on screening questions and data extraction are available in the Supplement.

## 3. Results

To ensure that all reviewers had the same understanding of the inclusion and exclusion criteria, we performed a consistency check, using *N* = 20 papers across 6 reviewers—CR, HK, MS, TMGB, RKB, and CPS (the same 20 papers for all the reviewers) in both the abstract and full-text stage. The kappa value obtained was 0.55 [31]. This statistic varies between −1 and 1, indicating 1 an almost perfect agreement. This level is qualitatively considered as ‘fair’. However, disagreements were present in papers that were going to be discarded either way because they did not pass the screening. After the results were known, a meeting was held to clarify the reasons for the disagreement and therefore improve the understanding of the questions.

### 3.1. Screening

**Figure 1.**
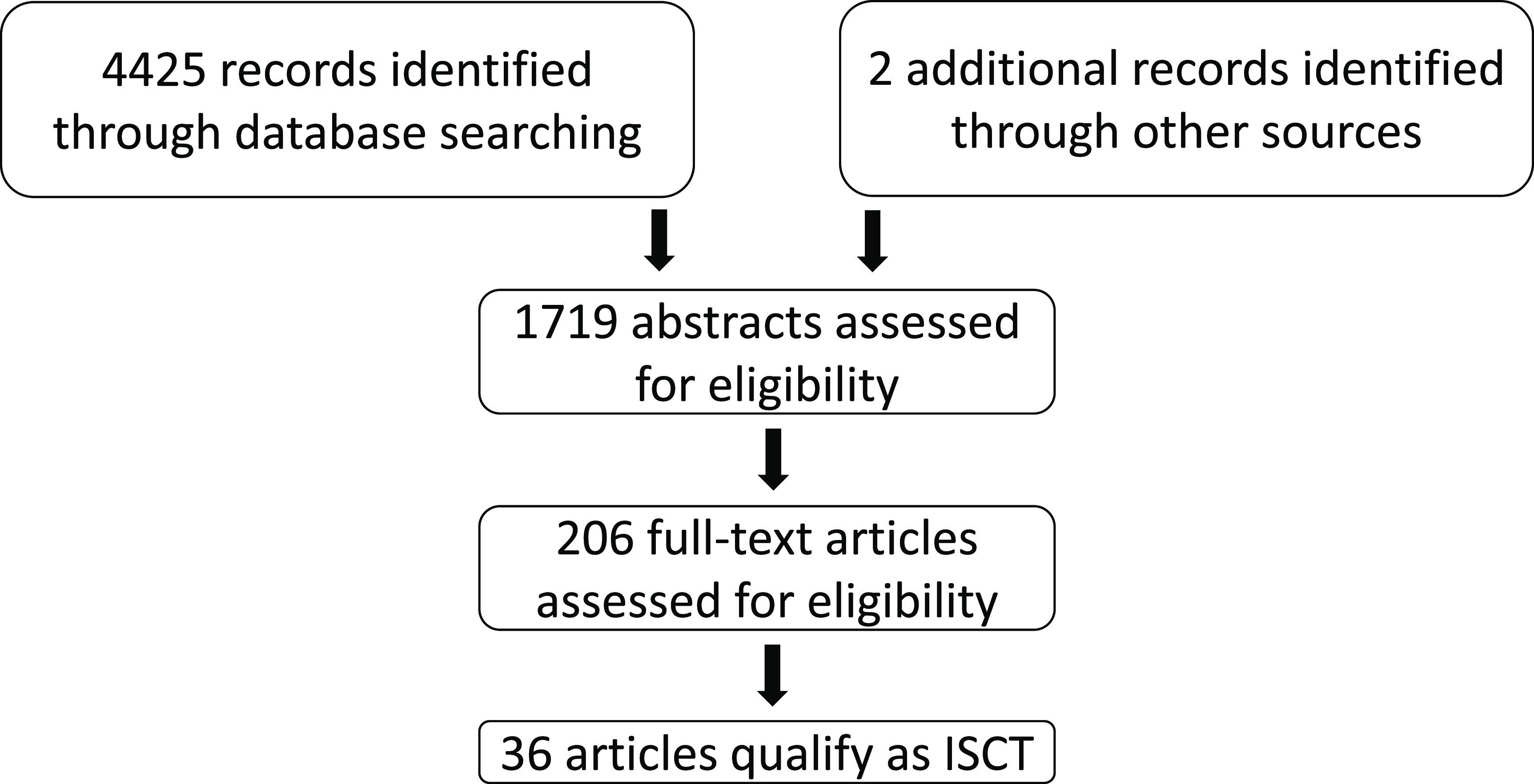
PRISMA flow chart of the systematic search strategy.

A summary of the screening process can be found in figure 1. A total of 4425 records were identified through the database search and 2 additional records were included by manual search. After removing duplicates, the 1719 abstracts were screened.

After the abstract screening, 1513 records were excluded, leaving 206 full-text articles evaluated for eligibility. From these, 161 articles were excluded. Details on the reasons can be found in the supplement. We also discarded 6 additional records that simulated an intervention in a single patient. We considered such studies to be digital twin studies and not an ISCT. This is comparable to the difference between a case study and a clinical trial. The 3 cases in which the authors used a representative cell model and then tested multiple drugs on it were also discarded because they did not involve the use of a cohort of virtual patients. The final 36 papers were analysed in this review.

### 3.2. Study summary

In the past decade, there has been an increase in published ISCTs, with 3 studies in 2013, increasing to 6 in 2021.

The first authors are predominantly from the US (13 studies), UK (11 studies), and China (4 studies). Taking into account that all the authors 96, 88, and 27 were affiliated with institutions in the US, UK, and Germany, respectively.

We analysed if any of the co-authors of the papers are affiliated with a company or if in the funding, disclosure or acknowledgements sections, they mentioned any help or funding from industrial partners. A total of $42\%$ of the ISCTs receive this type of industrial support (including but not limited to funding from AstraZeneca [10, 69], Biotronik [36], CSL Behring [71], Actelion Pharmaceuticals [22] or Abbott [55]).

#### 3.2.1. Modelling details

In general, there were three main types of physics that are used in ISCT: electrophysiology (EP), mechanics, and hemodynamics. Most studies ($75\%$) conducted hemodynamic simulations, while mechanics and EP are less modelled ($25\%$ and $22\%$, respectively). Note that a study can perform more than one type of modelling.

**Figure 2.**
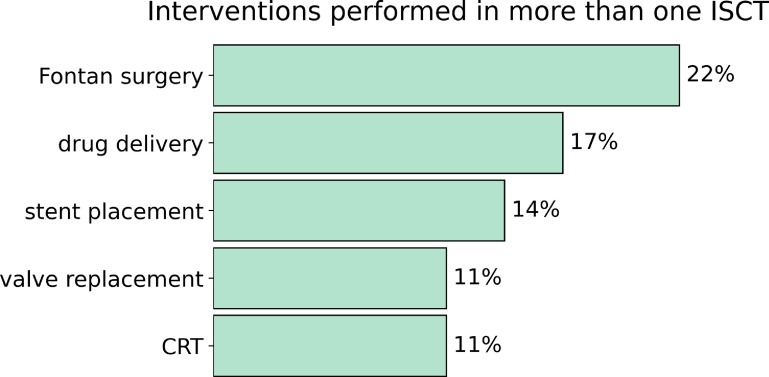
Interventions modelled in more than one of the ISCTs analysed. CRT stands for cardiac resynchronization therapy.

ISCTs were more likely to include models of the left ventricle and aorta ($25\%$ of the studies). The intervention modelled in these cases range from CRT, to ablation or stent placement. We analysed the interventions modelled across all ISCTs, resulting in a total 15 interventions, covering drugs, surgeries, and implant devices (figure 2).

**Figure 3.**
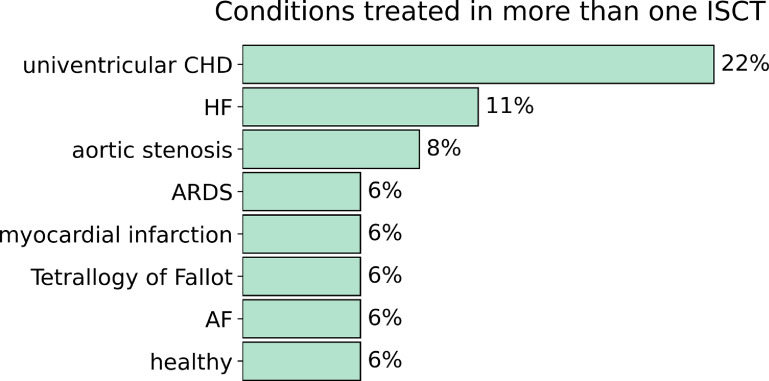
Conditions analysed in more than 1 ISCT. ‘CHD’ stands for congenital heart disease, ‘HF’ stands for heart failure, ‘AF’ stands for atrial fibrillation and ‘ARDS’ stands for acute respiratory distress syndrome.

The treated condition (see figure 3) was mainly univentricular congenital heart disease (CHD), in $22\%$ of the ISCTs, followed by heart failure in $14\%$ of the cases and aortic stenosis in $8\%$ of the cases. Acute respiratory distress syndrome was modelled in $6\%$ of the cases. This intervention was included because, although being pulmonary, the authors simulate the whole cardiovascular system measuring variables such as cardiac output. It is noteworthy, however, that $42\%$ of the articles analysed a condition that was not analysed in any other ISCT.

#### 3.2.2. Population analysed

In clinical trials, there is a formal process to determine the sample size based on the expected variability and the effect size of an intervention. The final sample size is then the number of patients recruited into the study. ISCTs can have models that map to a specific patient, synthetic models, or a mix of the two.

We made three distinctions in terms of the population used in the ISCT: if each virtual patient represents an actual subject (i.e. a digital twin), if the virtual patient is sampled from a population model (and, therefore, synthetic), or if it does not fit in any of the previous categories.

**Figure 4.**
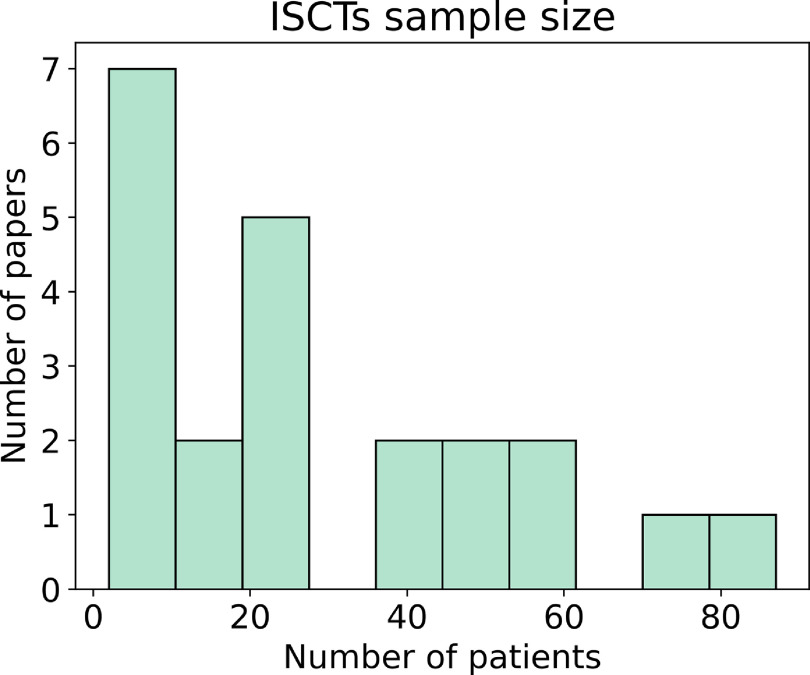
Distribution of the number of virtual patients used in the ISCTs analysed, where each virtual patient was the digital twin of a real patient.

In the $81\%$ of the studies in which the number of patients corresponds $1:1$ to specific patients, there was a range of 2–87 patients modelled with an average of 23 and a median of 12, see figure 4.

An alternative way of modelling is using population models, especially in pharmacokinetics and pharmacodynamics. In these scenarios, parametric distributions are created based on the clinical data of real patients. The simulations are then run using sampled parameters from these distributions. In $11\%$ of the studies analysed, this strategy was followed. In these cases, the number of virtual patients where the trial is conducted is considerably higher: 150 in [22], 1000 in [71], 1213 in [40] and $120~000$ in [10].

In 3 studies, the virtual patients did not fit any of the previous categories. In [28], atrial meshes of 2 patients are combined with the torso meshes of 8 different patients. Therefore, even if the atria and torsos are digital twins on their own, the final combined 16 virtual patients are synthetic. In [24], the cohort consists of 50 ventricular digital twins and 2 templates from statistical shape models (based on 493 and 600 digital twins). Lastly, in [69], the virtual patient cohort of 4 subjects is not made up of digital twins, but representative cases of the diseases of interest.

### 3.3. Reporting of patient demographics

In a clinical trial, patient demographics are essential and standard data to present. Although the level of information may vary between studies, we consider sex/gender, age, weight/body mass index, ethnicity and comorbidities to be basic demographic data.

When using a population model, we considered the demographics of the population used to build the model. In 2 of the 4 studies, no information is provided directly [10, 40], while in [22] and [71] sex, age and body weight were reported. In [71], ethnicity and comorbidities were also reported.

**Figure 5.**
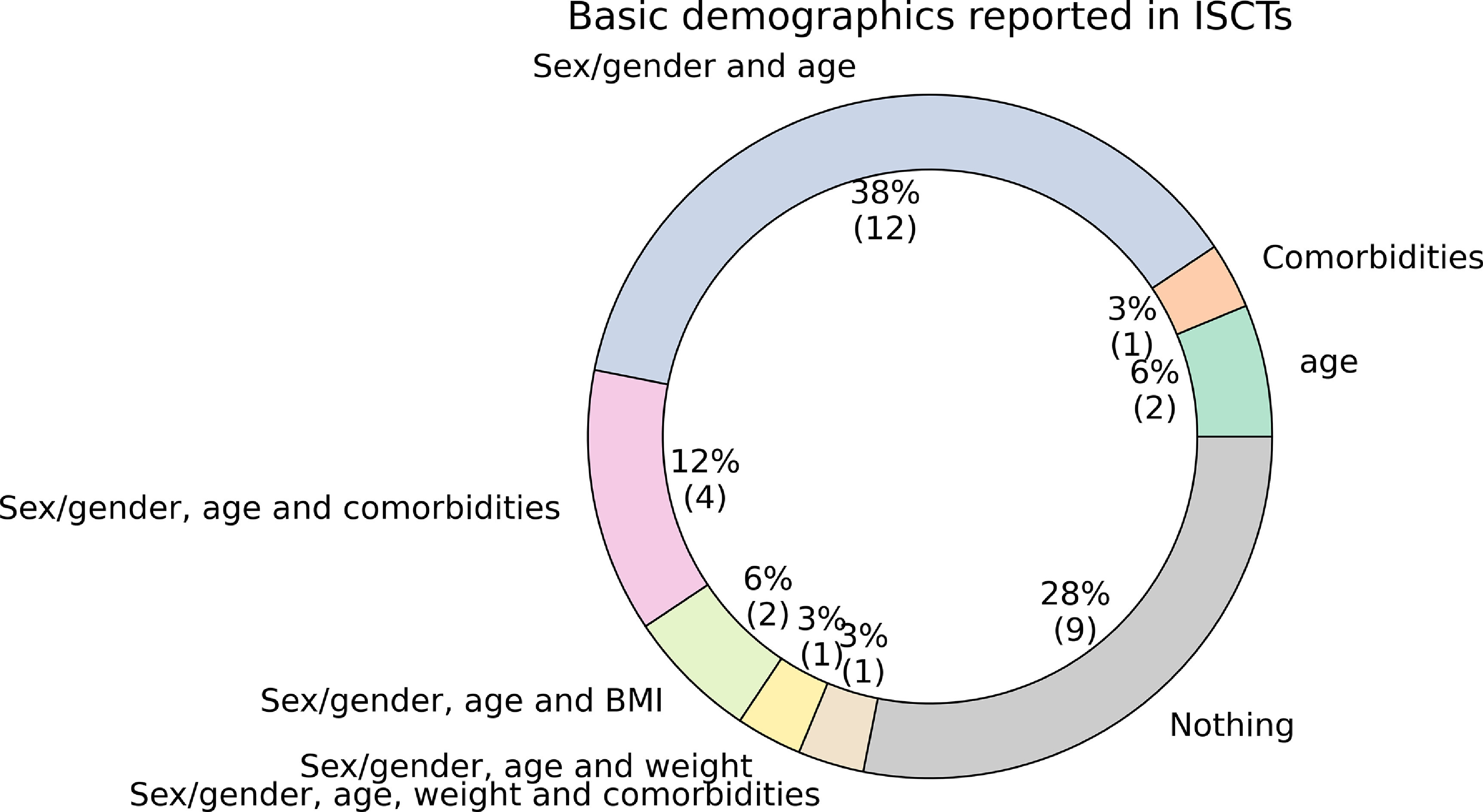
Basic demographics reported by the ISCTs, excluding studies where a population model is used. Comorbidities, sex/gender, age, body-mass index (BMI), weight and ethnicity were considered.

Excluding studies in which a population model is used, the basic demographic data for the population were not reported by $28\%$ of the reviewed ISCTs papers. In $38\%$ of the ISCTs, only the sex/gender and age of the population were reported. None of the ISCTs reported ethnicity. See figure 5 for the percentage of ISCTs reporting each combination of demographic information.

Misrepresentation of women is an ongoing problem in clinical trials, along with the lack of analysis of sex-disaggregated data [27]. In the cases where it was reported, we have analysed the percentage of virtual patients representing women involved in each study.

In population modelling studies, we can consider the percentage of women present in the patient cohort used to build the model. A total of $36\%$ and $89\%$ of the cohort were women in [71] and [22], respectively.

**Figure 6.**
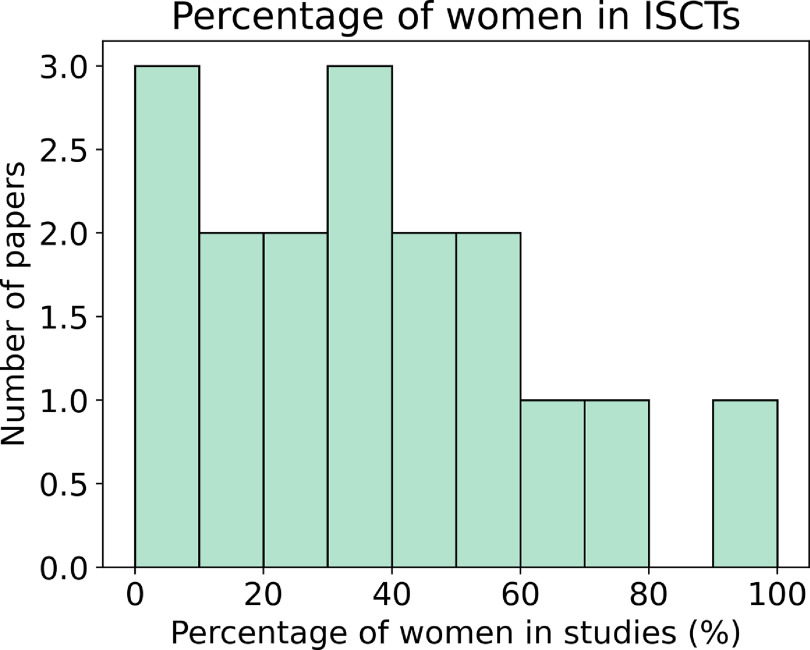
Percentage of women included in the ISCTs, when the population has more than 5 patients, excluding the use of population modelling studies.

Excluding population modelling studies, if we aggregate all patients from all studies that report this demographic information (*n* = 583), $32\%$ of the virtual patients were women. If we do not aggregate the data, we find that the studies had an average and a median of $40\%$ and $36\%$ of women, respectively. If we consider only studies with *n* > 5, this percentage decreased to $32\%$ and $35\%$, respectively. Figure 6 shows the distribution of the percentage of women represented in ISCTs with a population of more than 5 patients.

### 3.4. Reliability of the simulations

For all stakeholders (such as clinicians, regulators, or patients) to be confident in model-based clinical decisions, ISCTs must demonstrate to be reliable and trustworthy, reducing as much as possible the uncertainty in predictive capacity. Two of the main techniques for demonstrating the validity of a model are validation and uncertainty quantification.

There can be several degrees of validation, from comparing directly with patient measurements to qualitatively comparing the overlapping of the statistical distribution of some output of the simulations. Some of the authors, as in [14, 43, 60] reported that they used solvers, tools, or approaches that were previously validated. However, even if the validation of the tools used in the pipeline is an essential step, it does not guarantee the validation of the whole study. In $75\%$ of the studies, some kind of validation was performed, although the specific type of validation varied between the studies. For example, in [53], the authors compared the results of the computational fluid dynamics (CFD) simulations with *in vitro* data. In [3], the authors simulated the hemodynamic effects of occluding a fistula at various positions. As a validation step, the simulation results were compared with the actual surgery. However, as stated in the limitations, there was not good agreement. In [28], a machine learning algorithm was trained to classify simulated drivers of atrial fibrillation located in the pulmonary vein versus other types of drivers. The model was validated by being tested on clinical data, but the simulations were not.

In terms of uncertainty quantification, the most common approach was through a sensitivity analysis (SA) of the most influential parameters selected by the authors or across all model parameters. This SA could be local (small perturbations around the baseline) or global (effects on the variance of the results with large perturbations). However, this step was often omitted and $81\%$ of the studies did not provide any kind of SA. When they did, in $43\%$ of the cases, it involved a local SA [10, 25, 55].

### 3.5. Reproducibility of ISCTs

In a typical simulation study, clinical data are often used. Data can be used to fit the model parameters or as the domain of the simulation, for example, by creating meshes from imaging data. We analyse whether the clinical data used was first described in the article, whether it was taken from the literature, or whether the elements used derived from clinical data (such as meshes) are built into the study or used from a previous study. In $78\%$ of the cases analysed, the data were first described (and/or generated) in the study, as in [17, 58]. In the rest of the cases, the data were taken from previous studies, as in [1, 6, 68].

**Figure 7.**
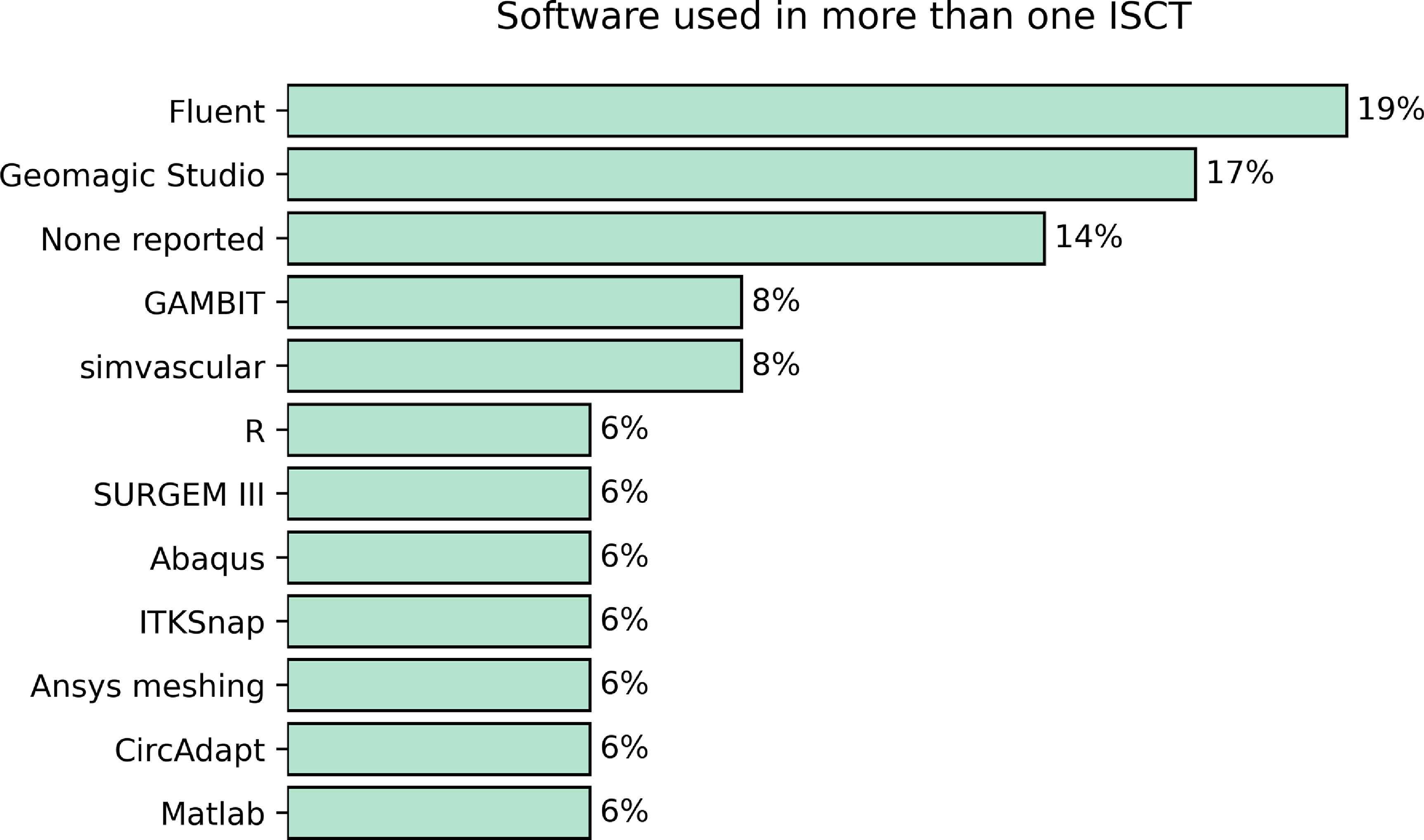
Most commonly used software or programming languages reported in more than one ISCT.

We analysed the software used (including programming languages) in ISCTs. This can be useful to know if there is a dominant software over the rest, or if researchers are not reporting the tools being used. Reporting on the software or programming language used in some of the steps of the study is more common. ANSYS FLUENT (ANSYS, Inc. Canonsburg, PA, USA) was the most widely used software in ISCTs, followed by Geomagic Studio. We note that in $14\%$ of the cases, the authors did not provide any information on the software they used. In figure 7 we show the most commonly used software in ISCTs.

Lastly, we analysed whether the articles were open access and the data availability statement. Of the articles analysed, $89\%$ of them were open access. However, there was more diversity in the data availability statement. A total of 31 out of 36 articles did not have a data availability statement or claimed that data deposition was not applicable to the article. Three of the studies claimed that the data was available on request [34, 52, 71]. In [70], the authors stated that the data would be available from the authors. Data and code were available in [10].

## 4. Discussion

There is a lack of structure and standard reporting in ISCTs, affecting several key aspects such as terminology, resources, and data sharing. There is a need to correct this situation and avoid potential biases and a reproducibility crisis.

### 4.1. Added value of ISCTs

### 4.2. The interaction between clinically relevant questions and widespread use software

We found that most of the studies ($75\%$) conducted hemodynamic simulations. It is well known in the community that the systems of equations describing fluid dynamics suffer from stability, convergence, and in some cases accuracy issues. It is therefore reasonable to think that the reason why more CFD simulations are being performed is not because it is a simple approach. Two factors should be taken into account to further understand this: the condition (and treatment) modelled and the software used.

Although the most prevalent cardiac condition worldwide is ischaemic heart disease [63], we found that the condition mostly modelled in ISCTs is univentricular CHD, while the intervention is Fontan surgery (in $22\%$ of the cases). This is in agreement with the extensive fluids simulations being performed. Moreover, when we analysed the software used we found that different ANSYS modules are the preferred options, ANSYS Fluent being used in $19\%$ of the cases. This information suggests that so far a robust, user-friendly, and documented software can have more impact in the development of ISCTs than purely pathophysiological or medical reasons.

ISCTs can provide extra value to both translational and clinical research. Quantifying the impact of ISCTs is never easy and there is not a systematic way to do it. As a first approach, we looked within the articles that cite the articles included in the review to check if there were any guidelines based on their work or any patent filed after their work. Only two works had a direct impact at the moment of this review. There was a patent filed after the work of [13] related to an operation scenario flow and mechanical modelling and analysis system of cardiovascular repair operations for newborn and foetus [42]. In terms of guidelines, the 2021 ESC Guidelines on cardiac pacing and cardiac resynchronization therapy recognised as an extra body of evidence the work by [25] on sex-dependent QRS guidelines.

One of the main potential advantages of ISCTs is cost reduction, since device/drug development life cycles are long and expensive, and failures at trial stage (and post-market) are not uncommon [51].

In [4], although they do not perform a cost-effectiveness analysis, they recognise this as a limitation. In other cases, it is mentioned that *in-silico* trials are a cost-effective alternative or complement to experiments [40, 66]. Cost-effectiveness has also been used as motivation in [6], to justify the use modelling, and in [25] to motivate the use of simplifications in the model. None of the papers reviewed performed a specific cost-effectiveness analysis.

A second advantage is to provide measurements and predictions of quantities and biomarkers that are either too invasive or simply cannot be measured directly clinically. Pressure and stress are two clear examples of this fact. Pressure measurements are usually obtained either indirectly (cuff pressure) or in a very invasive manner (pressure catheter in an artery or a cardiac chamber). This limits the number of clinical studies where direct, absolute pressure can be used as a biomarker. In ISCTs, however, this is a common biomarker when performing CFD simulation. Examples from the reviewed papers include [34].

In other cases, it is not physically possible to measure a biomarker, as is the case of stress. Once again, this can be commonly found in ISCTs, particularly in the studies running mechanics simulations. For instance, in [17] the authors quantify wall shear stress, in [36] endothelial shear stress or in [4], where they measure from the simulations both pressures and stresses.

### 4.3. Classifications of ISCTs

One of the main challenges we found in this review was the lack of a common vocabulary. Even in the papers that we identified as ISCTs, that terminology was not explicitly used. We need to be aware that the terminology of ‘clinical’ within ISCTs can be misleading, especially from the regulatory point of view, since ISCTs are not technically clinical trials. However, we decided to use the denomination of ISCTs mainly because we are focused on studies that use human data. Since human trials are often called clinical trials, we decided to adhere to this denomination. Nevertheless, we acknowledge that this is an early stage of the field, and this nomenclature could change as the field develops.

However, this problem is not specific to computational cardiology, but comes from the concept of *in-silico* medicine. As noted by Pappalardo *et al* [38], even the concept of *in-silico* medicine is not rigorously defined, sometimes referring to a method of experimentation (in line with *in vitro* and *in vivo*) and sometimes referring to the translational aspect of a computer simulation.

In the case of clinical trials, the definition is clear and subclassifications have arisen, such as the phases of a clinical trial or the type of a clinical trial (double-blind, cross-arm, randomised control trials, etc). For ISCTs, such distinctions do not exist yet and, as such, the terminology of ISCT is not standardised.

To establish a rigorous subclassification of ISCTs, we must take into account that there are additional types of trials compared to clinical trials. For example, a work in which the authors use a statistical model to increase the population size [10] does not fit neatly into any of the clinical classifications. Moreover, concepts such as blind/double-blind do not apply. Lastly, to move to the next phase of a clinical trial, the authors must have found a positive and statistically significant effect of the therapy or procedure tested. In ISCTs, since an arbitrarily large number of simulations could be run, significance metrics such as p-values can lose their meaning [45, 67]. Only significance tests should be considered where the number of results was increased by the addition of new patients, rather than by the number of simulations on a fixed number of patients.

From this analysis, we could differentiate ISCT studies depending on the level of personalisation, the interaction of the ISCT with a clinical trial, and whether the intervention has been performed on a physical patient, among others.

Depending on the level of personalisation, an ISCT could have patient- or population-based variability. In the first case, as in [1], the variability of the study comes from the different digital twins incorporated in the study, while in the latter case it comes from parameter sampling of a statistical distribution. There are hybrid cases, where virtual patients are created by combining parts of different digital twins [28] or by combining digital twins with statistical shape models [25].

Depending on the interaction of the ISCT with a clinical trial, we can make at least three distinctions. In the first case, the simulations are performed before an intervention or a clinical trial, to test effectiveness or safety, or to facilitate experimental design, for example. Multi-fidelity approaches [26, 41] could become very useful in these cases, where simulations (since they are cheaper than recruiting patients in a clinical trial) could be used to reduce the number of patients recruited in a subsequent clinical trial. In the second case, the ISCT is performed as a step of a clinical trial. For example, in [34], the authors test the impact of the information provided by an ISCT on the decision-making of cardiologists. Although the whole study was not an ISCT, a part of it was. Lastly, the third case, where simulations and modelling are used to reproduce and/or augment data from previously published clinical trials, as in [9].

Depending on whether the intervention has been performed in the actual patient or in a model, we can talk about an ISCT with a physical or virtual intervention. That is, if the simulations were used to model the intervention and the consequences or only the consequences. For example, in [36] a device was implanted in patients and CFD used to assess endothelial shear stress. On the other hand, in [66] a virtual surgery was performed to explore potential hemodynamic improvements in patients with apicocaval juxtaposition.

Other classifications present in clinical trials, such as cross-sectional and longitudinal, can still be applied to ISCTs. Although most of the studies were cross-sectional, there were some cases such as [44] in which the authors analysed the same patients at two different time points, and therefore it would qualify as a longitudinal ISCT.

There are three types of studies that we have not considered as ISCTs. First, if the study only had one patient, as in [32], we consider it a digital twin study. This would be analogous to a case study in the context of clinical trials. Even if in a digital twin study a higher number of hypotheses can be tested compared to a case study, the level of extrapolation to the population is more limited compared to a clinical trial or an ISCT. Second, if the study was a clinical trial of a computational tool, as in [19]. Here, they conduct a clinical trial in which they perform ablation, either guided by a computational tool or based on cardiologists’ experience. In this case, the population is not the patients, but the cardiologists. As such, there is no *in-silico* comparison of the outcome of an intervention. Lastly, the cases where the authors used a representative cell model and then tested multiple drugs on it, as in [29]. Since these studies did not involve a cohort of virtual patients, we did not include them as trials.

### 4.4. Risks associated to ISCTs

In some ISCTs, the patients models are personalised to collected or literature data. Therefore, some of the translation issues in digital twin science reported in [7] are expected to be passed on to ISCTs. These issues are big data hazards, computational power needs, cybersecurity, data sharing issues, governance and regulatory issues, ethical issues, and professional barriers.

Big data hazards such as biases and lack of generalisability of findings are expected to be present in ISCTs. One of the main tools to avoid these is through external validation, whether against clinical data [2] or data from the literature [55]. Validating against the values of the literature, although more accessible, can also not be representative of any specific patient [49]. Since multiple average values of different patients under different setups could be aggregated for validation, it is very unlikely that this is a model of any specific person. Although it is commonly interpreted that this way the model gains robustness, without adequate uncertainty quantification, the model’s validity is limited. We understand that not every institution may have access to the appropriate clinical data to validate in the required time frames, and this is a known caveat in the community.

In some of the cases analysed, the data was made available to the community: clinical datasets, scripts to reproduce the simulations and to reproduce the figures as in [10] and the files required to perform the simulations in [15]. This is an essential step not only to improve reproducibility, but also to advance research, as different studies could be carried out on the same data set [45, 55]. The main problem here and one of the potential reasons on why some authors prefer to make the data ‘available upon request’ is that due to regulatory and privacy restrictions not all data can be made open access. It is important here to distinguish between ‘patient data’, which can be used to identify the person (in which case regulations such as General Data Protection Regulation, GDPR, do not allow sharing openly) and ‘human data’, data that originally was generated using patient data, but that cannot be used to identify the patient. One of the most obvious examples of this difference is meshes that are derived from imaging data. Although the imaging data is patient data and cannot be shared between institutions without appropriate governance processes, the anonymised computational domain, i.e. the meshes can often be uploaded to repositories and used by anyone.

In terms of ethics in the case of ISCTs, a major ethical concern is bias. In [61] Turner *et al* showed that in US clinical studies from 2000 to 2020 only $43\%$ reported the ethnicity of the participants. The ISCTs reviewed are mainly based on retrospective data, and if ethnicity was not recorded in the data collection phase, this bias spreads from the clinical to the *in-silico* arena. This is especially relevant in diverse communities such as the US or capital cities such as London, where the percentage of minorities included in the study may not be obvious. In the articles analysed, we only found one study [71] reporting the ethnicity of the patients that made up the population model, although the countries with the highest number of ISCTs are the US and the UK. A similar issue is present with respect to sex/gender. Although the percentage of papers reporting it is higher, the distribution of the percentage of women is not centred around $50\%$, see figure 6.

### 4.5. Lack of regulation

For clinical trials, there is the CONSORT checklist of information [30] to include when reporting a randomised trial to help standardise randomised clinical trials. Such standards do not yet exist for ISCTs. This problem comes from digital health technologies, in general, where there is a regulatory gap with respect to safety, efficacy, and ethical compliance [16].

In the position paper by Pappalardo *et al* [39] they argue along the same lines, focussing on ISCTs for medical devices. One of the main consequences of the lack of regulation is the presence of quality differences between countries when it comes to evaluating the credibility of ISCTs. A possible solution to this would be to develop an International Organisation for Standardisation (ISO) standard that is recognised by the FDA and harmonised in the EU regulatory system.

The closest form of regulation to date comes from the Avicenna Alliance^7^ 7
www.vph-institute.org/avicenna.html.. This initiative was created in 2013, creating consensus between academic, industrial, and regulatory partners. Through this initiative, a detailed research roadmap was produced for ISCTs [64], adding 36 recommendations for all relevant stakeholders to consider.

One of the challenges identified in the Avicenna Alliance roadmap [64] was the need to provide more reproducible outcome measures to reduce, refine, and partially replace preclinical and clinical trials. This problem is still present in ISCTs, and providing open-access to data and methodologies is an essential step for this to happen.

For instance, we showed how in $14\%$ of the ISCTs there is no information at all about the software used, highly limiting the reproducibility of the study. Even when it is reported, as is the case with several examples of meshing software, details about the methodology need to be included. If the methodology is not rigorous enough or not consistent enough between studies, this could lead to a large variance in mesh profiles between studies or between models. Such variance could compromise the accuracy and reliability of the results.

### 4.6. Need for uncertainty quantification

One of the professional barriers reported in [7] is the generation of trust in models. One of the main ways to improve the reliability of models is through validation and SA. There are multiple ways in which they can be performed, highly dependent on the amount of data available and the time and cost of the simulations.

The lack of validation in computational models has also recently been noted by [33] in the case of the modelling of aortic aneurysms. In that review, Mourato *et al* reported that only $12\%$ of the articles performed numerical validation with patient-specific *in vivo* data and $76\%$ did not present a meaningful validation process. Regarding uncertainty quantification and SA, one of the conclusions of [33] was that there is a lack of studies on the impact of several parameters of the numerical simulations. This fits what we found in this review regarding the lack of local or global SAs in $81\%$ of the studies analysed.

In [11] the authors suggest a system to report the different degrees of validation for a study using patient-specific models and virtual cohorts of patient-specific models. The system for virtual cohorts could be adapted for ISCTs. Using this system, ISCTs can be more self-aware of limitations in respect to validation credibility. Even if $75\%$ of the studies analysed reported some kind of validation, it varies between the studies. Because of this, a gradation system rather than a validated/unvalidated classification can be more useful for ISCTs.

A complementary way to generate trust is to combine a conventional clinical trial with modelling tools, such as [4], where the ISCT predictions were compared with clinical decisions. Similarly, combining ISCTs with physical and experimental evidence can help build stronger evidence, as one approach compensates for the limitations of the other [5]. These studies can help spread the understanding of computational modelling, as long as the corresponding validation and SAs are performed to ensure the robustness of the model.

### 4.7. Need to scale up

In population modelling ISCTs, the number of ‘patients’ analysed is usually in the order of hundreds [10, 40]. However, when an ISCT is performed with digital twins, only 5 studies had more than 50 patients [15, 18, 25, 56, 59]. This can be a severe limitation, as is reported in multiple of the articles analysed.

Recently, some authors have started to provide open access data sets, such as four-chamber healthy hearts [46] and heart failure [54], atrial models [35, 48], 12-lead electrocardiograms (ECGs) [12], cardiac and pulmonary vascular structures [62] or aortas [47]. This type of work can help to accelerate translation. Furthermore, by combining elements from different available cohorts [45], new analyses can be performed that can improve the quality of the research carried out, especially in ISCTs.

For example, in [65] the authors analyse the aortic root rupture of 3 patients during a TAVI procedure. One of the conclusions is that due to the low occurrence rate of these cases, it is not feasible at the moment to perform a systematic study of a large cohort. Data sharing in ISCTs has some power in this case, as well as for rare diseases, since by sharing the meshes or other materials from these small ISCTs a large cohort of multicentre, potentially international cases can be created.

The lack of openly accessible data is also strongly linked with the original data presented in the studies. We found that $78\%$ of the ISCTs do not use data from previous studies. Although it is good to use novel data to improve knowledge and potentially avoid biases by increasing the sample size and the diversity of the cohort, such a low percentage of reused work might be linked to the lack of open-access data. This link also works in the other direction, since we found that only one work provided the data and the code, creating a loop that can be broken as soon as new studies start providing more data.

### 4.8. Limitations

Limitations of this systematic review include the specific choice of search keywords and the years chosen.

At the design stage of the review we went through several iterations of keywords making sure that the relevant papers were included. Although other combinations of keywords are possible, we consider it unlikely that a research paper on this field does not use any of the combinations used. For instance, although ‘computational’ might seem restrictive, if that word is not used, we believe it is very likely that the word ‘simulation’ is used. If none of them is used, we believe that other combinations will be hard to happen.

Regarding the years chosen for the review, we constrained to the years 2012–2021. Earlier works might appear, and this review should not be taken as a historical perspective on the field. This project started in 2022, and therefore to make sure that we included whole years we limited the search to studies published before 2022.

## 5. Conclusions

ISCTs are being conducted in cardiology. However, the standardisation of ISCT methodology is still in its infancy. There is a need for community agreement on minimal reporting standards on patient demographics, accepted standards for ISCT cohort quality control, and increased model and data sharing.

## Data Availability

All data that support the findings of this study are included within the article (and any supplementary files). https://github.com/CEMRG-publications/Rodero_2023_ProgrBiomedEng [72].
